# Cytokine and Chemokine Production in Mice Inoculated with NVX-CoV2373 (Nuvaxovid^®^) in Comparison with Omicron BA.4/5 Bivalent BNT162b2 (Comirnaty^®^)

**DOI:** 10.3390/vaccines11111677

**Published:** 2023-11-02

**Authors:** Tetsuo Nakayama, Takashi Ito, Ryoka Ishiyama, Kazuhiko Katayama

**Affiliations:** 1 Laboratory of Viral Infection, Ömura Satoshi Memorial Institute, Kitasato University, Tokyo 108-8641, Japan; ito.takashi@kitasato-u.ac.jp (T.I.); katayama@lisci.kitasato-u.ac.jp (K.K.); 2 Department of Pediatrics, Kitasato University Hospital, Sagamihara 252-0329, Japan; 3 Graduate School of Infection Control Sciences, Kitasato University, Tokyo 108-8641, Japan; di21001@st.kitasato-u.ac.jp

**Keywords:** NVX-CoV2373, BNT162b2, cytokine, chemokine, adverse events

## Abstract

A recombinant SARS-CoV-2 spike protein vaccine (NVX-CoV2373) has been licensed and has a lesser incidence of adverse events. To know the immunological mechanisms of adverse events, the production of cytokines and chemokines was investigated in mice inoculated with NVX-CoV2373. Serum IL-6 was detected on Day 1 of the first and second doses and the IFN-γ, IL-4, IL-10, TNF-α, and IL-6 levels increased on Day 1 of the second dose at the inoculation site. The enhanced production of the inflammatory chemokines (CCL2), homeostatic chemokine (CXCL13), and Th2 chemokine (CCL17) was observed at the inoculation site on Day 1 of the second dose. These findings were compared with data obtained following inoculation with BNT162b2 bivalent vaccine containing omicron BA.4/5. Significantly lower levels of inflammatory chemokines were detected on Day 1 after the first dose of NVX-CoV2373 in sera and inoculation site than those following inoculation with bivalent BNT162b2 (*p* < 0.01), reflecting a lower incidence of adverse events after immunization with NVX-CoV2373 in humans. NVX-CoV2373 induced significantly higher concentrations of IFN-γ, TNF-α, and IL-10 at the inoculation site obtained on Day 1 of the second dose (*p* < 0.05). Significant higher levels of Th2 chemokines, CCL11 and CCL17, were induced at the inoculation site on Day 1 of the second dose (*p* < 0.01) and they explain the booster IgG EIA antibody response after the second dose of NVX-CoV2373.

## 1. Introduction

A serious outbreak of unknown pneumonia was reported in Wuhan on 31 December 2019. The pathogenic agent was identified within several days and, soon after, a full-length nucleotide sequence was disclosed. The pathological agent was identified and named severe acute respiratory syndrome coronavirus-2 (SARS-CoV-2) later, due to its morphological features [1]. It spread to the EU and US within a few months. Although the case fatality rate was approximately 2–3% lower than that of SARS and Middle East respiratory syndrome (MERS), the transmission rate of SARS-CoV-2 was extremely high, threatening the public health [2]. Therefore, the WHO announced a pandemic alert on March 11, 2020, and encouraged the development of effective vaccines [1,2,3]. At the end of 2020, mRNA vaccines (BNT162b2 and mRNA1273) and an adeno virus-vectored vaccine (ChAdOx1) were licensed for emergency use [4,5,6].

Although mRNA vaccines have been used worldwide with high efficiency, local and systemic adverse events are common, for example, local pain at the injection site in 70–85% of cases; febrile reactions in 10–20% of cases; and headaches and fatigue at 40–60% [4,5]. After immunization with BNT162b2, pain at the injection site was reported among 83% of recipients aged 16–55 years after the first dose and 78% following the second dose. Five percent of people suffered from local redness or swelling. As for systemic adverse reactions, febrile illness was noted in 4% and 16% of the population after the first and second doses, respectively. More frequently observed adverse events were fatigue (47%), and headaches (42%) after the first dose, and fatigue (42%) and headaches (52%) following the second dose [4]. After immunization with mRNA-1273, solicited local pain at the injection site was noted in 86.9% and 89.9% of people following the first and second doses, respectively. For systemic adverse events, 0.9% and 17.4% of people had fever, 35.3% and 62.8% had a headache, and 38.4% and 67.6% had fatigue after the first and second doses, respectively [5]. Severe-grade systemic reactogenicity was more frequently observed after the second dose than it was after the first dose, although local reactogenicity occurred after both doses at high frequency. The adverse events were generally mild or moderate and less common among older adults. Local pain at the injection site might be related to inflammatory cytokine production, and serum inflammatory cytokines are associated with systemic adverse events of febrile illness. The cytokine profiles of mice inoculated with BNT162b2 and mRNA-1273 were investigated. Th1 and Th2 cytokines were detected at the inoculation site and in the serum samples after the second dose. mRNA-1273 induced higher levels of Th1 and Th2 cytokines than BNT162b2 did. IL-6 and G-CSF were also detected at the inoculation site on Day 1 of the first dose, but the levels decreased after Day 3, and enhanced production was demonstrated on Day 1 of the second dose [7]. The induction of inflammatory cytokines in the mouse model reflects the immunogenicity and adverse reactions observed in humans immunized with mRNA vaccines.

RNA is a ligand for the RNA sensor of TLR 3, 7, and 8, inducing type I IFN. In particular, double-stranded RNA triggers TLR3 signaling and activates retinoic acid-inducible gene (IRIG-I) or melanoma differentiation-associated gene 5 (MDA-5) to recruit caspases at the caspase recruit domain (CARD). Caspase induces the inflammatory cytokines IL-1β, IL-6, and TNF-α from pro-inflammatory cytokines [8,9]. RNA is a fragile molecule, and mRNA molecules encoding SARS-CoV-2 spike protein are embedded in lipid nanoparticles (LNP), consisting of phospholipids, cholesterol, polyethylene glycol, and ionizable cationic lipids to stabilize mRNA vaccines. The ionizable cationic lipids make a complex with negatively charged mRNA to transport into the endosome [10,11]. mRNA, itself, performs adjuvant activity through RNA sensor, inducing IFN-α/β for activating cytotoxic T lymphocyte (CTL), and LNPs contribute to stimulate inflammasome to regulate antibody responses through Th1 or Th2 cytokines.

For conventional SARS-CoV-2 vaccines, NVX-CoV2373 (Nuvaxovid^®^) was developed by Novavax Ltd. (Gaithersburg, MD, USA) using SARS-CoV-2 spike protein nanoparticles expressed by the baculovirus expression system with Matrix-M1 adjuvant. From the results of a phase III clinical trial, it showed sufficient protective effects: vaccine efficacy to prevent symptomatic COVID-19 was 89.7% (95% CI; from 80.2 to 94.6) after two doses. As for the local adverse events, the incidence of tenderness was reported in 53.3% and 76.4% of people after the first and second doses, respectively. Pain at the injection site for the first dose was found in 29.3% and in 51.2% of people following the second dose. The most common systemic adverse events were headache, muscle pain, and fatigue after both the first dose (24.5%, 21.4%, and 19.4%, respectively) and the second dose (40.0%, 40.3%, and 40.3%, respectively) [12]. The incidence of febrile reaction increasing body temperature ≥ 38 °C was reported in 2.0% of the population after the first dose and in 4.8% after the second dose. Generally, NVX-CoV2373 causes lower incidence of adverse events, in comparison with that of mRNA vaccines. In the present study, the induction levels of cytokines and chemokines were compared in mice inoculated with NVX-CoV2373 and BNT162b2.

## 2. Materials and Methods

### 2.1. Experimental Protocol

**Figure 1:**
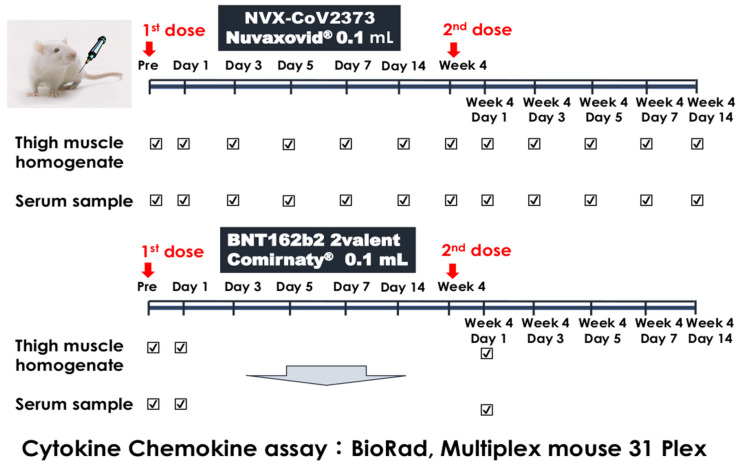
Experimental schedule. Five-week-old female BALB/c mice were inoculated with 0.1 mL of NVX-CoV2373 at the left thigh. Serum and thigh muscle homogenate samples were obtained from three mice for each time point and after inoculation with bivalent BNT 162b2, serum and thigh muscle samples were obtained before injection and on Day 1 of first and second doses.

Five-week-old female BALB/c mice were purchased from SLC Japan (Hamamatsu, Shizuoka Prefecture, Japan) and were inoculated with 0.1 mL of NVX-CoV2373 at the left thigh. Serum and thigh muscle samples were obtained from three mice for each time point on a scheduled day, as shown in Figure 1. The second dose was given at 4 weeks after the first dose at the same thigh region, and the following samples were obtained at the same time points. After inoculation with Omicron BA.4/5 bivalent BNT162b2, serum and thigh muscle samples were obtained before injection and on Day 1 of the first and second doses because peak cytokines were observed at this time-point in a previous report [7]. The study protocol was approved by the Ethical Committee of Animal Research of Kitasato University (approval number: 22-023).

### 2.2. Cytokine and Chemokine Assay

Approximately 120 mg of thigh muscle tissue at the injection site was obtained and homogenized as in a previous report [7], and serum samples were also stocked. The following mouse cytokines and chemokines were measured using Bio-Plex Pro^TM^ Mouse Chemokine Panel 31 plex (Bio-Rad Laboratories Inc., Hercules, CA, USA): BCA-1, CTACT, ENA-78, Eotaxin, Eotaxin2, Fractalkine, GM-CSF, I-309, I-TAC, IFN-γ, IL-1β, IL-2, IL-4, IL-10, L-16, IP-10, KC, MCP-1, MCP-3, MCP-5, MDC, MIP-1α, MIP-1β, MIP-3α, MIP-3β, RANTES, SCYB16, SDF-1α, TARC, and TNF-α.

### 2.3. IgG EIA Antibody against SARS-CoV-2 Spike Protein

Development of antibodies against SARS-CoV-2 spike protein were compared following inoculation with NVX-CoV2373 and BNT162b2. Stocked serum samples following inoculation with original BNT162b2 were used [7]. IgG antibodies against the SARS-CoV-2 spike protein (S) prototype were measured using #DK20-COV4E-S coated with 10 ng/well provided by Denka, Co., Ltd. (Tokyo, Japan) according to the manufacturer’s protocol. Briefly, serum was diluted 1:200 with the sample dilution buffer and incubated on the coated microplate for 1 h at room temperature. The wells were washed three times, and then anti-mouse IgG antibody conjugated with HRP (Jackson ImmunoResearch Laboratories Inc., Baltimore, MD, USA) was added and incubated at room temperature for 1 h. The substrate solution was added and incubated at room temperature for 30 min. The stop solution was then added (0.1 mL per well), and absorbance was measured at 450 and 630 nm using an iMark^TM^ microplate reader (Bio-Rad Laboratories Inc.).

### 2.4. Statistical Analysis

Cytokine and chemokine concentrations were analyzed, and box and whisker plots were created with the median titer and lower and upper range of 5–95%. Significance (*p* < 0.05) was determined by the Mann–Whitney U test using BellCurve for Excel 4.05 (Social Survey Research Information Co., Ltd., Tokyo, Japan).

## 3. Results

### 3.1. Cytokine Production Profiles following Inoculation with NVX-CoV2373

**Figure 2:**
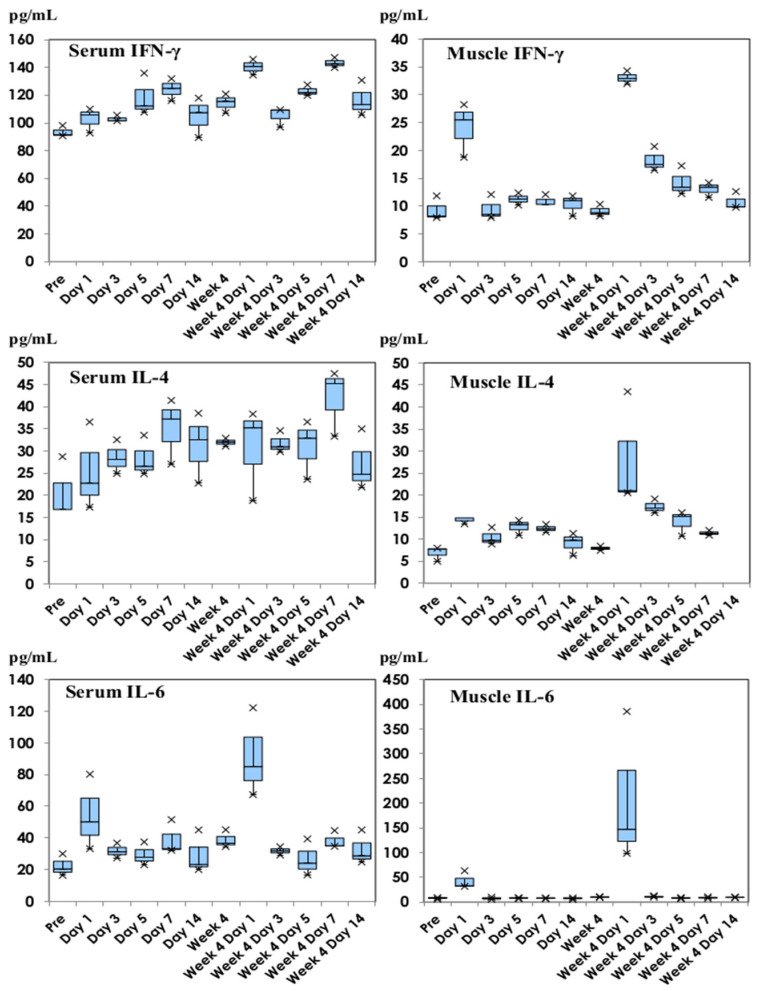
Cytokine profiles of IFN-γ, IL-4, and IL-6 following inoculation with NVX-CoV2373. IFN-γ, IL-4, and IL-6 production profiles in serum and thigh muscle homogenate samples are shown, representing Th1, Th2, and inflammatory cytokines. They are shown in box and whisker plots with median titers and the range of 5–95%.

The cytokine production profiles of IFN-γ, IL-4, and IL-6 are shown in Figure 2, representative of Th1 (IFN-γ) and Th2 cytokines (IL-4). IL-6 is a well-known inflammatory cytokine and functionally regulates the production of antibodies. The level of IFN-γ production increased in the thigh muscle homogenate samples on Day 1 of the first dose and was enhanced after the second dose, with a gradual decrease later. The level of serum IFN-γ gradually increased after inoculation. However, the serum IL-4 level increased after inoculation, similar to the kinetics of serum IFN-γ production. The amount of IL-4 slightly increased after the first dose and was enhanced on Day 1 of the second dose in thigh muscle homogenate. A similar trend was noted for IL-10 production. As for inflammatory cytokine production, IL-6 was transiently produced both in serum and thigh muscle homogenates on Day 1 of the first dose and was enhanced on Day 1 of the second dose. TNF-α production showed a similar profile to IL-6 production.

### 3.2. Chemokine Production Profiles following Inoculation with NVX-CoV2373

**Figure 3:**
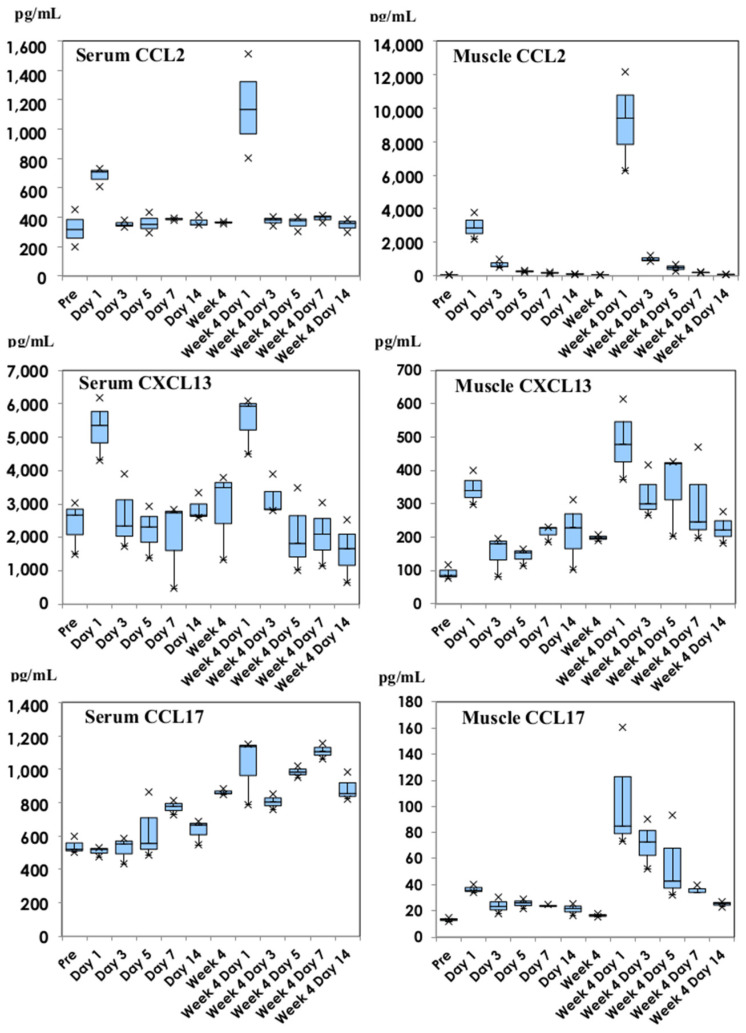
Chemokine concentrations of CCL2, CXCL13, and CCL17 in serum and thigh muscle homogenate samples following inoculation with NVX-CoV2373. CCL2, CXCL13, and CCL17 are representative of inflammatory, homeostatic, and Th2 chemokines, respectively. They are shown in box and whisker plots with median titers and the range of 5–95%.

Chemokines are divided into three functionally different types: inflammatory, homeostatic, and Th2 chemokines [13,14]. Kinetics of chemokines are shown in Figure 3.

CCL2 (monocyte chemotactic protein: MCP-1), which is representative of inflammatory chemokines, was produced on Day 1 of the first dose, and the level enhanced on Day 1 of the second dose both in serum samples and thigh muscle homogenates. As for the same inflammatory chemokines, CCL3 (macrophage inflammatory protein: MIP-1α) and CCL5 (regulated on activation, normal T-cell expressed and secreted: RANTES) were also induced in thigh muscle homogenates on Day 1 of the first and second doses, showing a similar profile to that of CCL2.

The Homeostatic chemokine, CXCL13 (B cell-attracting chemokine: BCA-1) and Th2 chemokine, CCL17 (thymus and activation-regulated chemokine: TARC), are also shown in Figure 3. In both the serum and thigh muscle homogenates, CXCL13 was detected on Day 1 of the first and second doses, with a gradual decline in quantity after Day 3 of the second dose. The CCL17 concentration was higher in serum after the second dose and was induced in the thigh muscle homogenates on Day 1 of the second dose, with a subsequent decline.

### 3.3. Comparison of Cytokines following NVX-CoV2373 and Bivalent BNT162b2

**Figure 4:**
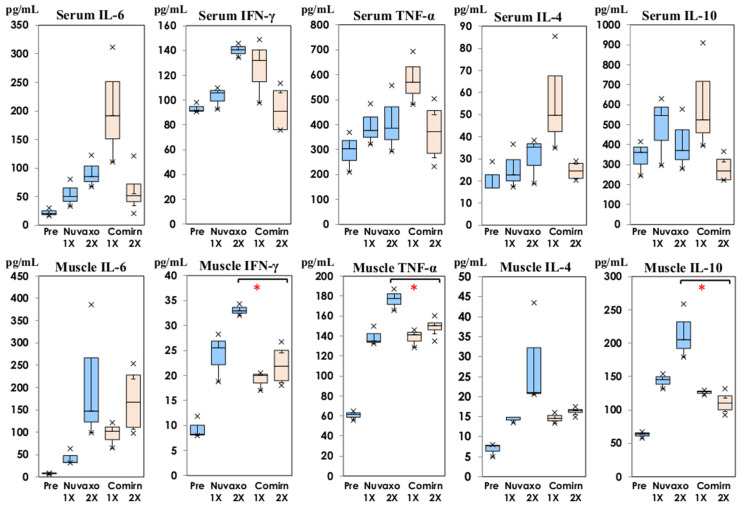
Comparison of IL-6, IFN-γ, TNF-α, IL-4, and IL-10 in serum and thigh muscle homogenates on Day 1 of the first and second doses following NVX-CoV2373 (Nuvaxovid^®^) and bivalent BNT162b2 (Comirnaty^®^). Cytokine concentrations are shown in box and whisker plots with median titers and the range of 5–95%: NVX-CoV2373 in blue columns (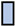: Nuvaxovid^®^) and orange columns (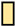: Comirnaty^®^). * *p* < 0.05.

The cytokine production amounts on Day 1 of the first and second doses were compared following inoculation with NVX-CoV2373 and bivalent BNT162b2, and the results are shown in Figure 4.

The serum IL-6, IFN-γ, TNF-α, and IL-4 levels were lower on Day 1 of the first inoculation with NVX-CoV2373, but they were not significant. Those on Day 1 of the second dose were similar concentrations in serum samples following inoculation with NVX-CoV2373 and bivalent BNT162b2. IFN-γ, TNF-α, and IL-10 in thigh muscle homogenates showed significantly higher levels on Day 1 of the second dose following NVX-CoV2373 than following inoculation with bivalent BNT162b2 (*p* < 0.05).

### 3.4. Comparison of Chemokines following NVX-CoV2373 and Bivalent BNT162b2

**Figure 5:**
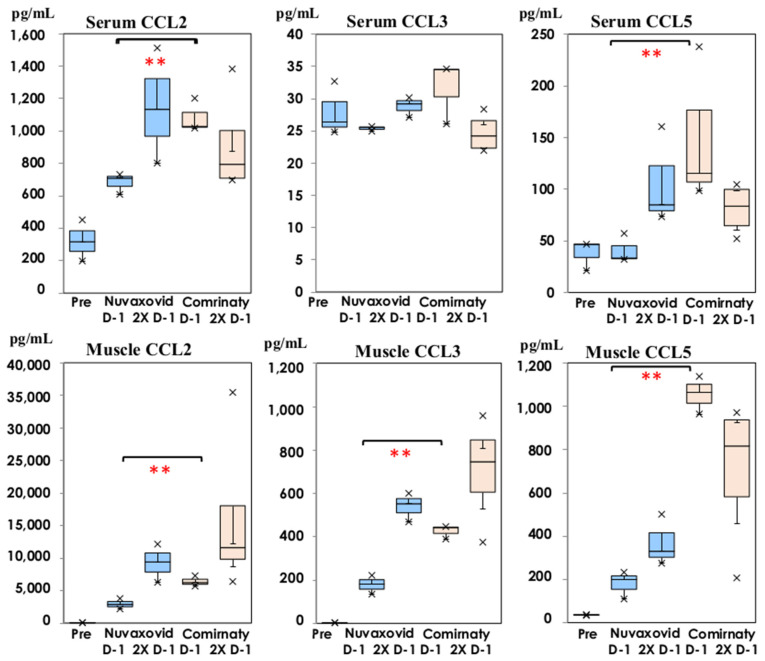
Comparison of CCL2, CCL3, and CCL5 in serum and thigh muscle homogenates on Day 1 of the first and second doses following NVX-CoV2373 (Nuvaxovid^®^) and bivalent BNT162b2 (Comirnaty^®^). Chemokine concentrations are shown in box and whisker plots with median titers and the range of 5–95%: NVX-CoV2373 in blue columns (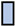: Nuvaxovid^®^) and orange columns (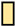: Comirnaty^®^). ** *p* < 0.01.

The inflammatory chemokines in serum and thigh muscle homogenates were compared following inoculation with NVX-CoV2373 and bivalent BNT162b2, and the results are shown in Figure 5.

The serum CCL2 and CCL5 levels were significantly lower on Day 1 of the first dose following NVX-CoV2373 than those following inoculation with bivalent BNT162b2 (*p* < 0.01). The levels of inflammatory chemokines, CCL2, CCL3, and CCL5, were significantly lower in the thigh muscle homogenates at the injection site following NVX-CoV2373 on Day 1 of the first dose than those following bivalent BNT162b2 inoculation (*p* < 0.01).

**Figure 6:**
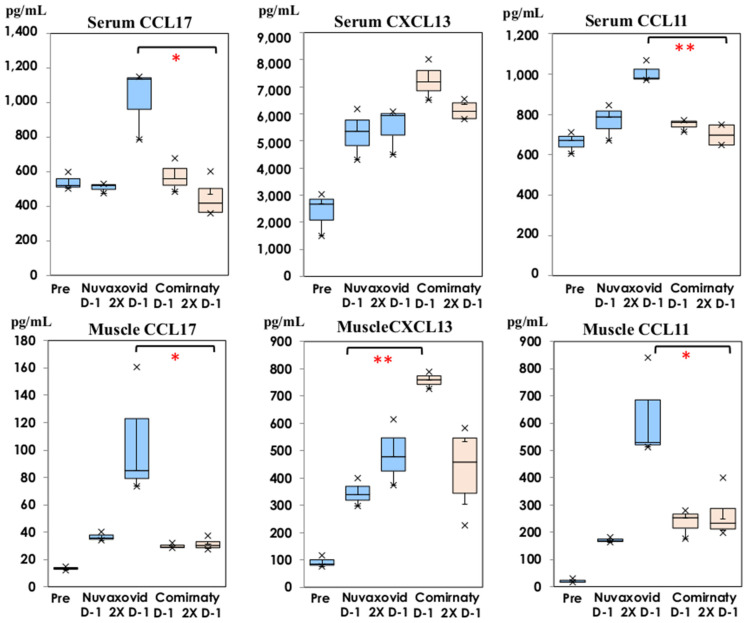
Comparison of CXCL13, CCL17 and CCL11 in serum and thigh muscle homogenates on Day 1 of the first and second doses following NVX-CoV2373 (Nuvaxovid^®^) and bivalent BNT162b2 (Comirnaty^®^). Chemokine concentrations are shown in box and whisker plots with median titers and the range of 5–95%: NVX-CoV2373 in blue columns (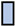: Nuvaxovid^®^) and orange columns (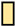: Comirnaty^®^). * *p* < 0.05, ** *p* < 0.01.

The comparison of homeostatic and Th2 chemokines following NVX-CoV2373 and bivalent BNT162b2 are shown in Figure 6. The level of CXCL13 was higher in thigh muscle homogenates on Day 1 of the first dose of bivalent BNT162b2 than it was following NVX-CoV237 inoculation (*p* < 0.01), but it was not significant in the sera. CCL17 and CCL11 showed significantly higher levels following inoculation with NVX-CoV2373 than with BNT162b2 on Day 1 of the second dose both in the serum and thigh muscle homogenates at the inoculation site (*p* < 0.05, or *p* < 0.01).

### 3.5. Development of IgG EIA Antibodies against SARS-CoV2 Spike Protein

**Figure 7:**
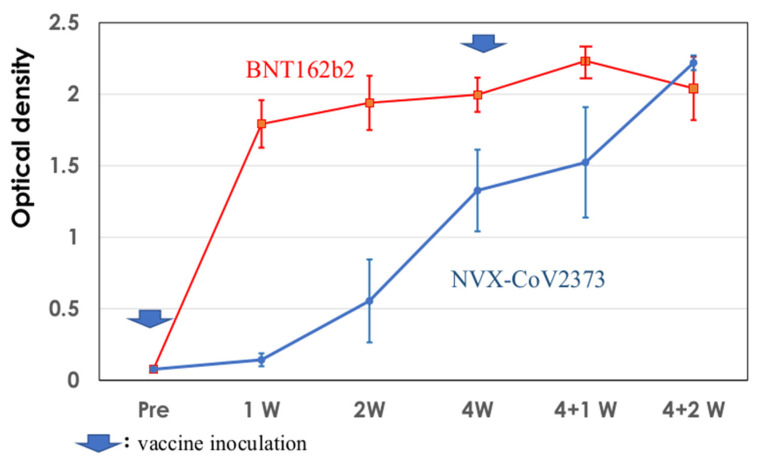
IgG EIA antibodies’ response against SARS-CoV-2 spike antigen following immunization with NVX-CoV2373 and BNT162b2 in a mouse model. Three mice were inoculated with NVX-CoV2373 and BNT162b2, and the second dose was given 4 weeks after the first dose. EIA titers are shown as the mean ± 1.0 SD of optical density.

The serum IgG EIA antibodies against SARS-CoV 2 spike protein were measured using the serum samples taken before inoculation, at 1, 2, and 4 weeks, and the again at 1 and 2 weeks after the second dose of NVX-CoV2373 in the present study; additionally, they were measured in the stocked serum samples following inoculation with BNT162b2 in a previous study [7]. The results are shown in Figure 7. Elevated levels of IgG EIA antibodies were detected one week after the first dose of BNT162b2, showing similar levels afterward. After inoculation with NVX-CoV2373, low levels of IgG antibodies were detected 2 weeks after the first dose and increased after the second dose. They assumed similar levels to one week after the first dose of BNT162b2.

## 4. Discussion

Vaccine induces specific immune responses to confer protection against infection or to mitigate the disease severity upon subsequent exposure. Cellular and humoral immune responses develop through non-specific innate immune responses. The development of antibodies binds pathogens or pathogenic agents and cellular immunity, especially CTL, eliminates the virus-infected cells to prevent further expansion of pathogenic agents. This involves collaboration between cytokines and chemokines. These reactions are triggered through pattern recognition receptors: pathogen-associated molecular patterns (PAMPs) and damage-associated molecular patterns (DAMPs), inducing cytokines and chemokines for trafficking to the draining lymph node [13,14,15]. They closely modulate the development of cellular and humoral immune responses, and, conversely, are linked to the occurrence of vaccine-adverse events [16,17].

We investigated cytokine production in vaccine recipients with a febrile illness within 48 h after immunization with single or different combinations of DPT, IPV, Hib, PCV, and Rota vaccines. Significantly higher levels of human G-CSF were detected in sera obtained from vaccine recipients with a febrile illness than in those without febrile reactions [18]. Higher IL-1β levels were produced in the lymphocyte cultures obtained from non-immunized infants when they were stimulated with PCV rather than DPT or Hib, and the concurrent stimulation, including PCV7, enhanced the production of IL-1β [18].

To elucidate the relationship between the increased cytokines and adverse events, inflammatory nodules were observed in muscle tissues at the injection site and were found to have been infiltrated with polymorph neutrophils following inoculation with adjuvanted vaccines [19]. The autolysis of migrated neutrophils caused neutrophil extracellular traps (NETs) in inflammatory nodules [19]. Host cellular DNA, reactive oxygen species (ROS), and endogenous substances are released through NETs and activate DAMPs. Released DNA has been shown to have other effects to localize the inflammation. Long-term cytokine production was examined for PCV, Hib, DTaP, bivalent HPV (Cervarix^®^), and four-valent HPV (Gardasil^®^) [19]. DTaP and PCV induced IL-6 and G-CSF in thigh muscle homogenates on Day 5 of the first dose. Cytokine inducibility depends on the biological activity of the adjuvants in the vaccine additives. A conventional aluminum adjuvant was used for DPT, PCV, and Gardasil^®^. ASO4 adjuvant containing monophosphoryl lipid A (MPL) and aluminum is used for the adjuvant system of Cervarix^®^ and they induced higher levels of inflammatory cytokines of IL-6 and G-CSF following the first and second doses [19]. MPL is a ligand of TLR4, and aluminum adjuvant stimulates inflammasome to induce the production of inflammatory cytokines [20,21].

Following this line of research, we investigated the induction of cytokines after inoculation with varicella zoster virus vaccine (Shingrix^®^), using AS01_B_ [22]. The AS01_B_ adjuvant system contains MPL, *Quillaja saponaria* Molina extract QS-21, cholesterol, and dioleoylphosphatidylcholine (DOPC), forming liposomes [23]. It induced similar profiles of IL-6 and G-CSF in thigh muscle homogenates and serum IFN-γ, IL-6, IL-5, and IL-10 were induced at higher levels on Day 1 of the first and second doses in mice inoculated with Shingrix^®^ [24]. Although a high incidence of local pain at the injection site was noted with headache, malaise, and febrile illness following immunization with Shingrix^®^, it induced high vaccine efficacy to prevent the occurrence of varicella zoster and post-zoster neuralgia ≥ 95% [22,25]. Inflammatory cytokines trigger the CD4 helper functions of Th1 and Th2 responses and are also associated with adverse reactions on the different viewpoints [16,17].

mRNA vaccines BNT162b2 and mRNA-1273 were licensed with marked vaccine efficacy, but a high incidence of local and systemic adverse events was reported [4,5]. mRNA was recognized by pattern recognition receptors, an RNA sensor of TLR3, 7,8, inducing IFN-α/β, and LNP stimulates the inflammasome [8,9,10,11]. Cytokine production was investigated in a mouse model [7]. Th1, Th2, and inflammatory cytokines were produced at higher levels at the injection site on Day 1 of the second dose than they were after the first inoculation with BNT162b2 or mRNA-1273. mRNA-1273 induced higher levels of Th1 and Th2 cytokines than BNT162b2 [7]. These findings can explain the higher incidence of local adverse events after a second dose [4,5].

NVX-CoV2373 was authorized for emergency use and lower incidences of local pain and systemic adverse reactions were reported [12,26] compared with those in the phase III reports of BNT162b2 and mRNA1273 [4,5]. The enhanced production of several cytokines and chemokines was observed on Day 1 of the second doses [7]. Then, we compared cytokine and chemokine production following inoculation with NVX-CoV2373 and bivalent BNT162b2 in the present study. Serum IL-6 and TNF-α showed a lower tendency on Day 1 of the first inoculation with NVX-CoV2373 in Figure 4, but their differences were not significant. The primary function of IL-6 and TNF-α is to up-regulate the multiple inflammatory chemokines [15]. A significantly lower level of inflammatory chemokines (CCL2, CCL3, and CCL5) was detected on Day 1 of the first dose in the serum and thigh muscle homogenates inoculated with NVX-CoV2373 than that of the mice inoculated with bivalent BNT162b2 (*p* < 0.01) in Figure 5, but there was no difference on Day 1 of the second dose. Monocytes and inflammatory cells migrate to areas demonstrating more inflammatory chemokines such as CCL2, according to the different concentrations [27]. CCL5 mediated the trafficking and homing of lymphoid cells, being associated with a wide range of inflammatory disorders [28]. These inflammatory chemokine profiles explain the lower incidence of local adverse events following immunization with NVX-CoV2373.

Contrarily, significantly higher levels of IFN-γ, TNF-α, and IL-10 were detected on Day 1 of the second dose in thigh muscle homogenates inoculated with NVX-CoV2373. Significantly higher levels of Th2 chemokines (CCL17 and CCL11) were induced in thigh muscle homogenates on Day 1 of the second dose, and the same finding was noted in the sera (*p* < 0.01). Higher levels of CXCL13 were induced on Day 1 of the first dose following inoculation with bivalent BNT162b2. CXCL13 was originally considered to be a B cell chemoattractant involved in the recruitment of B cells. Recently, CXCL13 binds to a receptor of CXCR5 expressed on CD4 + T cells. The association of CXCL13/CXCR5 modulates T-B interaction, being a key mediator of adaptive humoral immune responses [29]. CCL17 binds to CCR4 expressed on Th2 lymphocytes, recruiting these cells to inflammation sites [30]. It was induced through GM-CSF, and the GM-CSF-CCL17-CCR4 axis is considered to have a broad biological function related to inflammatory pain [25]. In the present study, GM-CSF was measured. There was no significant difference in the serum samples; however, GM-CSF at the injection site following inoculation with NVX-CoV2373 was 6.14 ± 1.32 pg/mL and 10.43 ± 2.35 pg/mL on Day 1 of the first and second doses (<0.5 pg/mL on Day 0), respectively. In comparison with those following inoculation with BNT162b2, GM-CSF on Day 1 of the second dose following BNT162b2 showed significantly higher levels of 21.37 ± 4.66 pg/mL (*p* < 0.05). These cytokine and chemokine networks induce inflammation and recovery, and modulate the development of acquired immune responses [15,16].

The kinetics of the development of serum IgG EIA antibodies against SARS-CoV-2 spike protein showed different profiles. BNT162b2 induced marked levels of EIA antibodies as early as one week after the first dose; however, detectable EIA antibodies developed two weeks after the first dose, and the level increased after the second dose of NVX-CoV2373. Similar EIA IgG antibody development was reported after mRNA vaccination, and a delay in the IgG response was reported following inoculation with NVX-CoV2373 [31,32,33]. This is related to the different profiles of cytokines and chemokines following inoculation with BNT162b2 and NVX-CoV2373, with higher levels of CXCL13 after first dose of inoculation with BNT162b2 and higher levels of Th2 chemokines (CCL17 and CCL11) in muscle tissues and sera on Day 1 of the second dose of NVX-CoV2373.

NVX-CoV2373 contains purified recombinant spike protein nanoparticles with Matrix-M^TM^ adjuvant. Matrix-M^TM^ is composed of 40 nm nanoparticles derived from *Quillaja saponaria* Molina, cholesterol, and phospholipid [34]. Matrix-M^TM^ is a mixture of Matrix-A and Matrix-C. The Saponin-based adjuvant was initially known as an immune-stimulating complex (ISCOM^®^) using Quil A as the saponin and generated both humoral and cellular immune responses. The ISCOM^®^ adjuvant was evaluated for use as human papilloma virus (HPV) or hepatitis C (HCV) vaccines but was not applied to further clinical studies because of pain/aches at the injection site [35]. AS01_B_ used in Shingrix^®^ and Matrix-M^TM^ in NVX-CoV2373 contain similar materials based on *Quillaja saponaria*. The IL-6 production profiles were investigated following inoculation with Shigrix^®^ and NVX-CoV237, showing different profiles of cytokines, i.e., marked levels of IL-6 in mice inoculated with Shigrix^®^. AS01_B_ used Shingrix^®^ is a saponin-based combined adjuvant with MPL. Through our experiments in a mouse model inoculated with current vaccines, it was found that the different profiles of cytokine and chemokine can explain the biological characteristics of vaccine adverse events and immunological responses.

In the near future, purified recombinant protein vaccines using a new adjuvant system, or mRNA vaccines other than COVID-19 will be developed and reactogenicity is a major concern. The findings obtained using our cytokine and chemokine production in mouse model show that inflammatory chemokines predict an adverse reaction in preclinical examinations.

## 5. Conclusions

NVX-CoV2373 induced lower levels of inflammatory chemokines on Day 1 of the first dose in the serum and thigh muscle homogenates at inoculation site in comparison with those caused by bivalent BNT162b2, reflecting the lower incidence of adverse events. Higher levels of Th2 chemokines were induced in both serum and thigh muscle homogenates at the inoculation site on Day 1 of the second dose of NVX-CoV2373 than those observed following bivalent BNT162b2 inoculation, and this may relate to booster immune responses after the second dose of NVX-CoV2373.

## Data Availability

The datasets used and analyzed during this study are available from the corresponding author upon reasonable request.

## References

[1] Li Q., Guan X., Wu P., Wang X., Zhou L., Tong Y., Ren R., Leung K.S.M., Lau E.H.Y., Wong J.Y. (2020). Early transmission dynamics in Wuhan, China, of novel coronavirus-infected pneumonia. N. Engl. J. Med..

[2] Wu Z., McGoogan J.M. (2020). Characteristics of and important lessons from the coronavirus disease 2019 (COVID-19) outbreak in China summary of a report of 72314 cases from the Chinese Center for Disease Control and Prevention. JAMA.

[3] Corey L., Mascola J.R., Fauci A.S., Collins F.C. (2020). A strategic approach to COVID-19 vaccine R&D. Science.

[4] Polack F.P., Thomas S.J., Kitchin N., Absalon J., Gurtman A., Lockhart S., Perez J.L., Marc G.P., Moreira E.D., Zerbini C. (2020). Safety and efficacy of the BNT162b2 mRNA COVID-19 vaccine. N. Engl. J. Med..

[5] Baden L.R., El Sahly H.M., Essink B., Kotloff K., Frey S., Novak R., Diemert D., Spector S.A., Rouphael N., Creech B. (2021). Efficacy and safety of the mRNA-1273 SARS-CoV-2 vaccine. N. Engl. J. Med..

[6] Voysey M., Clemens S.A.C., Madhi S.A., Weckx L.Y., Folegatti P.M., Aley P.K., Angus B., Baillie V.L., Barnabas S.L., Bhorat Q.E. (2021). Safety and efficacy of the ChAdOx1 nCoV-19 vaccine (AZD1222) against SARS-CoV-2: An interim analysis of four randomised controlled trials in Brazil, South Africa, and the UK. Lancet.

[7] Nakayama T., Sawada A., Ito T. (2023). Comparison of cytokine production in mice inoculated with messenger RNA vaccines BNT162b2 and mRNA-1273. Microbiol. Immunol..

[8] Yoneyama M., Fujita T. (2009). Recognition of viral nucleic acids in innate immunity. Rev. Med. Virol..

[9] Yu M., Levine S.J. (2011). Toll-like receptor 3, RIG-I-like receptors and the NLRP3 inflammasome: Key modulators of innate immune responses to double-stranded RNA viruses. Cytokine Growth Fact. Rev..

[10] Samaridou E., Heyes J., Lutwyche P. (2020). Lipid nanoparticles for nucleic acid delivery: Current perspectives. Adv. Drug Deliv. Rev..

[11] Ndeupen S., Qin Z., Jacobsen S., Bouteau A., Estanbouli H., Igyártó B.Z. (2021). The mRNA-LNP platform’s lipid nanoparticle component used in preclinical vaccine studies is highly inflammatory. iScience.

[12] Health P.T., Galiza E.P., Baxter D.N., Boffito M., Browne D., Burns F., Chadwick D.R., Clark R., Cosgrove C., Galloway J. (2021). Safety and efficacy of NVX-CoV2373 COVID-19 vaccine. N. Engl. J. Med..

[13] Chen K., Bao Z., Tang P., Gong W., Yoshimura T., Wang J.M. (2018). Chemokines in homeostasis and diseases. Cell Mol. Immunol..

[14] Graham G.J. (2009). D6 and the atypical chemokine receptor family: Novel regulators of immune and inflammatory processes. Eur. J. Immunol..

[15] Striz I., Brabcova E., Kolesar L., Sekerkova A. (2014). Cytokine networking of innate immunity cells: A potential target of therapy. Clin. Sci..

[16] Pollard A., Bijker E.M. (2021). A guide to vaccinology: From basic principles to new developments. Nat. Rev. Immunol..

[17] Nakayama T. (2016). An inflammatory response is essential for the development of adaptive immunity-immunogenicity and immunotoxicity. Vaccine.

[18] Kashiwagi Y., Miyata A., Kumagai T., Maehara K., Suzuki E., Nagai T., Ozaki T., Nishimura N., Okada K., Kawashima H. (2014). Production of inflammatory cytokines in response to diphtheria-pertussis-tetanus (DPT), haemophilus influenzae type b (Hib), and 7-valent pneumococcal (PCV7) vaccines. Hum. Vaccine Immunother..

[19] Nakayama T., Kashiwagi Y., Kawashima H. (2018). Long-term regulation of local cytokine production following immunization in mice. Microbiol. Immunol..

[20] Casella C.R., Mitchell T.C. (2008). Putting endotoxin to work for us: Monophosphoryl lipid A as a safe and effective vaccine adjuvant. Cell Mol. Life Sci..

[21] Stöver A.G., Correia J.D.S., Evans J.T., Cluff C.W., Elliott M.W., Jeffery E.W., Johnson D.A., Lacy M.J., Baldridge J.R., Probst P. (2004). Structure-activity relationship of synthetic Toll-like receptor 4 agonists. J. Biol. Chem..

[22] Chlibek R., Bayas J.M., Collins H., de la Pinta M.L.R., Ledent E., Mols J.F., Heineman T.C. (2013). Safety and immunogenicity of an AS01-adjuvanted varicella-zoster virus subunit candidate vaccine against herpes zoster in adults ≥50 years of age. J. Infect. Dis..

[23] Didierlaurent A.M., Laupèze B., Di Pasquale A., Hergli N., Collignon C., Garçon N. (2017). Adjuvant system AS01: Helping to overcome the challenges of modern vaccines. Expert Rev. Vaccines.

[24] Nakayama T., Sawada A., Ito T. (2022). Increased production of inflammatory cytokines after inoculation with recombinant zoster vaccine in mice. Vaccines.

[25] Oxman M.N., Levin M.J., Johnson G.R., Schmader K.E., Straus S.E., Gelb L.D., Arbeit R.D., Simberkoff M.S., Gershon A.A., Davis L.E. (2005). A vaccine to prevent herpes zoster and postherpetic neuralgia in older adults. N. Engl. J. Med..

[26] Masuda T., Murakami K., Sugiura K., Sakui S., Schuring R.P., Mori M. (2022). Safety and immunogenicity of NVX-CoV2373 (TAK-019) vaccine in healthy Japanese adults: Interim report of a phase I/II randomized controlled trial. Vaccine.

[27] Singh S., Anshita D., Ravichandiran V. (2021). MCP-1: Function, regulation, and involvement in disease. Int. Immunopharmacol..

[28] Appay V., Rowland-Jones S.L. (2001). RANTES: A versatile and controversial chemokine. Trends Immunol..

[29] Harrer C., Otto F., Radlberger R.F., Moster T., Pilz G., Wipfler P., Harrer A. (2022). The CXCL13/CXCR5 immune axis in health and disease—Implications for intrathecal B cell activities in neuroinflammation. Cells.

[30] Lee K.M.C., Jarnicki A., Achuthan A., Fleetwood A.J., Anderson G.P., Ellson C., Feeney M., Modis L.K., Smith J.E., Hamilton J.A. (2020). CCL17 in inflammation and pain. J. Immunol..

[31] Wisnewski A.V., Luna J.C., Redlich C.A. (2021). Human IgG and IgA responses to COVID-19 mRNA vaccines. PLoS ONE.

[32] Keech C., Albert G., Cho I., Robertson A., Reed P., Neal S., Plested J.S., Cloney-Clark S., Zhou H., Smith G. (2020). Phase 1–2 trial of a SARS-CoV-2 recombinant spike protein nanoparticle vaccine. N. Engl. J. Med..

[33] Mukhopadhyay L., Yadav P.D., Gupta N., Mohandas S., Patil D.Y., Shete-Aich A., Panda S., Bhargava B. (2021). Comparison of the immunogenicity & protective efficacy of various SARS-CoV-2 vaccine candidates in non-human primates. Indian J. Med. Res..

[34] Reimer J.M., Karlsson K.H., Lövgren-Bengtsson K., Magnusson S.E., Fuentes A., Stertman L. (2012). Matrix-M^TM^ adjuvant induces local recruitment, activation and maturation of central immune cells in absence of antigen. PLoS ONE.

[35] Pearse M.J., Drane D. (2005). ISCOMATRIX^®^ adjuvant for antigen delivery. Adv. Drug. Deliv. Rev..

